# Hepatitis C virus nonstructural protein 4B induces lipogenesis via the Hippo pathway

**DOI:** 10.1007/s00705-023-05743-4

**Published:** 2023-03-15

**Authors:** Chen Zou, Hongxi Tan, Jun Zeng, Minqi Liu, Guangping Zhang, Yi Zheng, Zhanfeng Zhang

**Affiliations:** 1 Department of Pathology, The First Affiliated Hospital, Jinan University, Guangzhou, 510630 China; 2 Center for Medical Experiments, University of Chinese Academy of Sciences-Shenzhen Hospital, Shenzhen, 518016 China; 3 Huadu District People’s Hospital of Guangzhou, Guangzhou, 510600 China; 4 Department of Laboratory Science, First Affiliated Hospital of Guangzhou University of Chinese Medicine, Guangzhou, 510600 China

## Abstract

Hepatitis C virus (HCV) infection causes abnormal lipid metabolism in hepatocytes, which leads to hepatic steatosis and even hepatocellular carcinoma. HCV nonstructural protein 4B (NS4B) has been reported to induce lipogenesis, but the underlying mechanism is unclear. In this study, western blots were performed to investigate the effect of NS4B protein levels on key effectors of the Hippo and AKT signaling pathways. Yes-associated protein (YAP) and moesin-ezrin-radixin-like protein (Merlin) are effectors of the Hippo pathway. NS4B downregulated Merlin and phosphorylated YAP (p-YAP) protein expression while increasing the expression of the key AKT pathway proteins p-AKT and NF-κB. By observing the levels of AKT pathway proteins when Merlin was overexpressed or silenced, it was determined that Merlin mediates the AKT pathway. We suggest that HCV NS4B may mediate the AKT signaling pathway by inhibiting the Hippo pathway. Lipid droplets were observed in Huh7.5 cells overexpressing NS4B, and they increased significantly in number when Merlin was silenced. Overexpression of NS4B and Merlin silencing enhanced the expression of sterol regulatory element binding proteins (SREBPs), which have been demonstrated to be key regulatory factors controlling fatty acid synthesis. NS4B and Merlin silencing also enhanced the *in vitro* proliferative capacity of hepatocellular carcinoma cells. In conclusion, NS4B induces lipogenesis via the effect of the Hippo-YAP pathway on the AKT signaling pathway and thereby plays a significant role in the pathogenesis of HCV-associated diseases.

## Introduction

Hepatitis C virus (HCV) is an enveloped positive-sense single-stranded RNA virus that causes chronic liver disease, cirrhosis, hepatocellular carcinoma (HCC), and other HCV-related liver diseases [1]. HCV infection is widespread throughout the world and is usually associated with high morbidity and mortality, as there is no effective medical treatment to contain HCV infection or inhibit its progress. It is believed that the pathogenicity of the virus and the intractability of HCV infection are primarily due to the ability of the virus to disrupt the regulation of host processes such as innate immunity and lipid metabolism. HCV has been linked to lipid and lipoprotein metabolism in clinical studies for many years [2, 3]. The efficiency of HCV RNA replication has been shown to be dependent on the accessibility of important lipid elements and membrane fluidity, which is positively correlated with host lipid metabolism [4, 5]. HCV infection reduces the synthesis of low-density lipoprotein (VLDL) in the host and slows down the oxidative decomposition of lipids in infected cells, which is conducive to HCV replication and pathogenicity [3].

HCV NS4B is a 27-kDa protein whose central domain consists of four transmembrane domains (TMs) and a Walker A nucleotide-binding motif. NS4B mainly functions in mediating viral replication and functions that affect replication, such as lipid metabolism [6]. Expression of NS4B alters the endoplasmic reticulum (ER) membrane and promotes the formation of a membranous web that serves as a scaffold for viral RNA replication. Mutations in the N-terminal region of NS4B can cause this protein to lose its ability to bind to lipid droplets, resulting in the abolishment of viral RNA replication and demonstrating the important role of NS4B in lipid metabolism and replication of HCV RNA [7]. Park and coworkers have shown that the HCV NS4B protein regulates SREBP1 through the AKT pathway. SREBP1 is a key endoplasmic-reticulum-binding transcription factor that controls lipogenesis and lipid uptake [8]. Furthermore, Hu et al. have shown that the PI3K/AKT pathway can be triggered by NS4B in HCC through upregulation of Snail protein synthesis and suppression of the Hippo signaling pathway [9].

The Hippo signaling pathway is evolutionarily conserved and modulates cell proliferation to control organ size in various species. Yes-associated protein (YAP) is one of the primary downstream effectors of the Hippo pathway [10]. Merlin (also known as neurofibromin 2, NF2) and Scrib participate in the Hippo signaling pathway as upstream effectors. It has been reported that 65–85% of HCC patients show increased YAP protein levels [11, 12]. By mimicking the effects of glypican 3 on CD81 expression, HCV activates the Hippo pathway in hepatocytes, resulting in an acceleration of the progression of hepatocellular carcinoma [13]. Hippo has been shown to affect the p53 signaling pathway to regulate SREPB1 activity at various levels and to modulate cholesterol and lipid levels [14].

Although HCV NS4B is known to have a significant effect on lipid-related metabolism, the pathways and mechanisms involved are unknown. In the present study, we found that HCV NS4B can induce lipogenesis via the Hippo-YAP and AKT signaling pathways by activating effector molecules, thereby contributing to the replication and pathogenicity of HCV.

## Materials and methods

### Cell lines and culture

The human hepatocyte cell line Huh7.5 and human embryonic kidney cell line HEK293T were purchased from CCTCC (Wuhan, China). The cells were cultured in DMEM (Gibco) supplemented with 10% (v/v) fetal bovine serum (Gibco, USA), 100 U of penicillin per mL, 25 mM NaHCO_3_ and 100 μg of streptomycin (Sangon Biotech, China) per mL. Cells were incubated in a 5% CO_2_ incubator at 37℃.

### Transient transfection

The entire NS4B gene was amplified by PCR and inserted into the plasmid pcDNA3.1 vector to construct the recombinant plasmid pcDNA3.1-NS4B. The pFLAG-Merlin plasmid was kindly provided by Prof. Huang Laiqiang (Graduate School at Shenzhen, Tsinghua University, China). HEK293T or Huh7.5 cells were seeded and cultured in 6-well plates, and when the cells reached about 80% confluence, they were transfected with the plasmid, using Lipofectamine 2000 (Invitrogen, Karlsruhe, USA) according to the manufacturer's instructions.

For siRNA transfection, about 5 × 10^5^ HEK293T or Huh7.5 cells were seeded in each well of a 12-well plate, cultured until they were about 60% confluent, and then transfected with an siRNA against Merlin or a control siRNA (si-control) using RNAiMAX (Invitrogen, USA). The cells were harvested after 48 or 72 h for further treatments or tests. The target sequence of the Merlin siRNA was 5ʹ-GAAACATCTCGTACAGTGA-3ʹ. The siRNA was purchased from Genepharm, China.

### Western blot analysis

Cells were harvested by centrifugation and washed twice with ice-cold phosphate-buffered saline (PBS). The cell pellets were lysed with cell lysis buffer (QIAGEN, Germany) on ice for 30 min, followed by centrifugation at 12,000 × *g* for 10 min to collect the supernatant, whose total protein concentration was determined using a BCA Protein Assay Kit (Thermo Scientific, USA). Then, 10 μg of protein was inactivated by boiling and subjected to SDS-PAGE and wet transfer to a PVDF membrane in a cold bath (Immobilon, USA). After blocking with 5% BSA for 2 h at room temperature to eliminate the effect of nonspecific binding, the membranes were incubated with specific primary antibodies for 4 h at room temperature or overnight at 4℃. Antibodies against NF-κB, Merlin, and NS4B were purchased from Abcam, UK, and the antibodies against AKT1/2/3, phosphorylated AKT, Yap, phosphorylated Yap, GAPDH, and SREBP-1 were purchased from Santa Cruz, USA. After washing three times with TBST, the membranes were incubated with HRP-conjugated secondary antibodies at room temperature for 2 h. Finally, the immunoblots were developed using ECL luminescence reagent (Sangon, China).

### Real-time quantitative PCR

Total RNA was extracted from transfected cells using TRIzol Reagent (Thermo Fisher, USA), and a RevertAid First Strand cDNA Synthesis Kit (MBI Fermentas, Germany) was used to reverse transcribe the RNA into cDNA. The primer sequences for PCR were as follows: SREBP-1 forward, 5’-GGAGCCATGGATTGCACATT-3’; SREBP-1 reverse, 5’-GGCCCGGGAAGTCACTGT-3’; CTGF forward, 5’-CGTGCCGGTGCCCGGACGAG-3’; CTGF reverse, 5’-GGCCGGGGAGCCGAAGTCAC-3’; β-actin forward, 5’-GTGGGGCGCCCCAGGCACCA-3’; β-actin reverse, 5’-CTCCTTAATGTCACGCACGATTTC-3’. The level of expression of each gene was estimated by the ΔΔCT method and normalized to β-actin.

### Oil Red O staining

Huh7.5 cells cultured for 24 h after transfection with a plasmid or siRNA were washed with PBS, and the cells were fixed by treatment with 10% formaldehyde for 10 min. After washing with PBS for 1 min with 60% isopropanol for 15 seconds to remove residual reagent, the cells were stained with Oil Red O solution in 40% water and 60% isopropanol) for 10 min at 37℃, washed twice with 75% alcohol, and washed three times with PBS. The cells were then counterstained with hematoxylin for 2 min and washed three times with PBS. The stained cells were sealed and photographed using a microscope (Olympus, Japan) at a magnification of 40×.

### Colony formation assay

Approximately 1500 Huh7.5 cells were inoculated onto methylcellulose medium in a 35-mm Petri dish and cultured at 37℃ in a 5% CO_2_ incubator. On the 14th day of culture, the cell colonies were fixed with methane-acetone (1:1) and stained with crystal violet, and the colonies were photographed, counted, and analyzed.

### Statistical analysis

Image J was used to analyze western blot results. Image Pro Plus 6.0 software was used to quantify Oil Red O staining results. GraphPad Prism 5.0 software (GraphPad Software, USA) was used for statistical analysis. The data are shown as the mean ± standard deviation (S.D.), and each experiment was repeated independently at least three times unless stated otherwise. Differences between two groups were assessed using Student’s *t*-test (two-tailed, unpaired). At *p* < 0.05, the results were considered to be statistically significant.

## Results

### NS4B downregulates Merlin protein expression and upregulates expression of key proteins of the AKT pathway

**Fig. 1:**
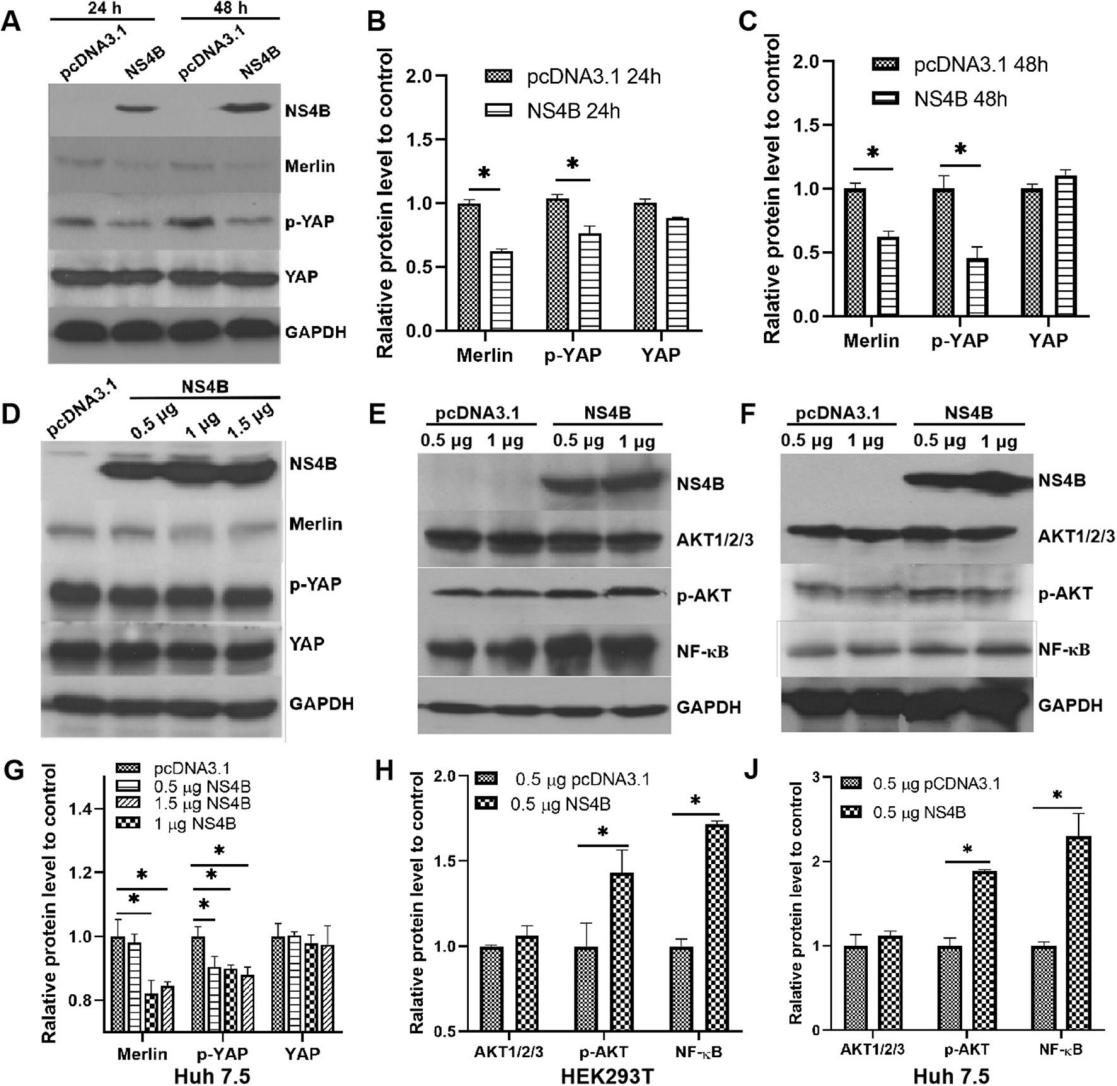
Downregulation of Merlin protein expression and upregulation of expression of key proteins of the AKT pathway by NS4B. (A-C) Huh7.5 cells were transfected with plasmid pCDNA3.1-NS4B to overexpress the NS4B protein, and plasmid pcDNA3.1 was used as a control. The Merlin, p-YAP, and YAP protein levels were estimated by western blot at 24 h or 48 h post-transfection. (D and G) Huh7.5 cells were transfected with 0.5, 1, or 1.5 μg of plasmid pCDNA3.1-NS4B to overexpress the NS4B protein, and plasmid pcDNA3.1 was used as a control. The Merlin, p-YAP, and YAP protein levels were analyzed at 24 h or 48 h post-transfection. (E and H) The relative amounts of AKT1/2/3, p-AKT, and NF-κB were estimated in HEK293T cells transfected with 0.5 μg of plasmid pCDNA3.1-NS4B to overexpress the NS4B protein, and 0.5 μg of plasmid pCDNA3.1 was used as a control. (F and J) Huh7.5 cells were treated the same as in panels E and H (*, *p* < 0.05).

To investigate the effect of NS4B on the Hippo-YAP signaling pathway, NS4B was overexpressed in Huh7.5 cells, which were collected and analyzed by western blot assay. As shown in Figure 1A, NS4B was successfully overexpressed, and the expression level increased with time. Western blots showed that NS4B overexpression reduced the expression of Merlin and the level of YAP phosphorylation (p-YAP) at both 24 and 48 hours post-transfection (Fig. 1A-C). When Huh7.5 cells were transfected with different amounts of NS4B plasmid for 24 hours, a positive correlation was observed between the amount of downregulation and the plasmid concentration used for transfection (Fig. 1D and G). NS4B, however, had no effect on the total YAP expression level.

We also studied the expression and phosphorylation level of key proteins of the AKT signal pathway by western blot assays. Total AKT1/2/3, phosphorylated AKT (p-AKT), and NF-κB protein levels were compared in HEK293T or Huh7.5 cells transfected with 0.5 μg or 1 μg pCDNA3.1-NS4B for 24 h. Whereas AKT1/2/3 protein expression showed little change, p-AKT and NF-κB protein levels were elevated significantly (Fig. 1G-L) in both HEK293T and Huh7.5 cells overexpressing NS4B, indicating that HCV NS4B modulates the AKT and Hippo-YAP pathways by phosphorylation of YAP.

### Merlin mediates the AKT signaling pathway

We hypothesized that the Hippo-YAP signaling pathway and AKT signaling pathway are connected by Merlin. To test this, the effects of different amounts of Merlin expression on the levels of total AKT1/2/3, p-AKT, and NF-κB protein were examined using a western blot assay.

**Fig. 2:**
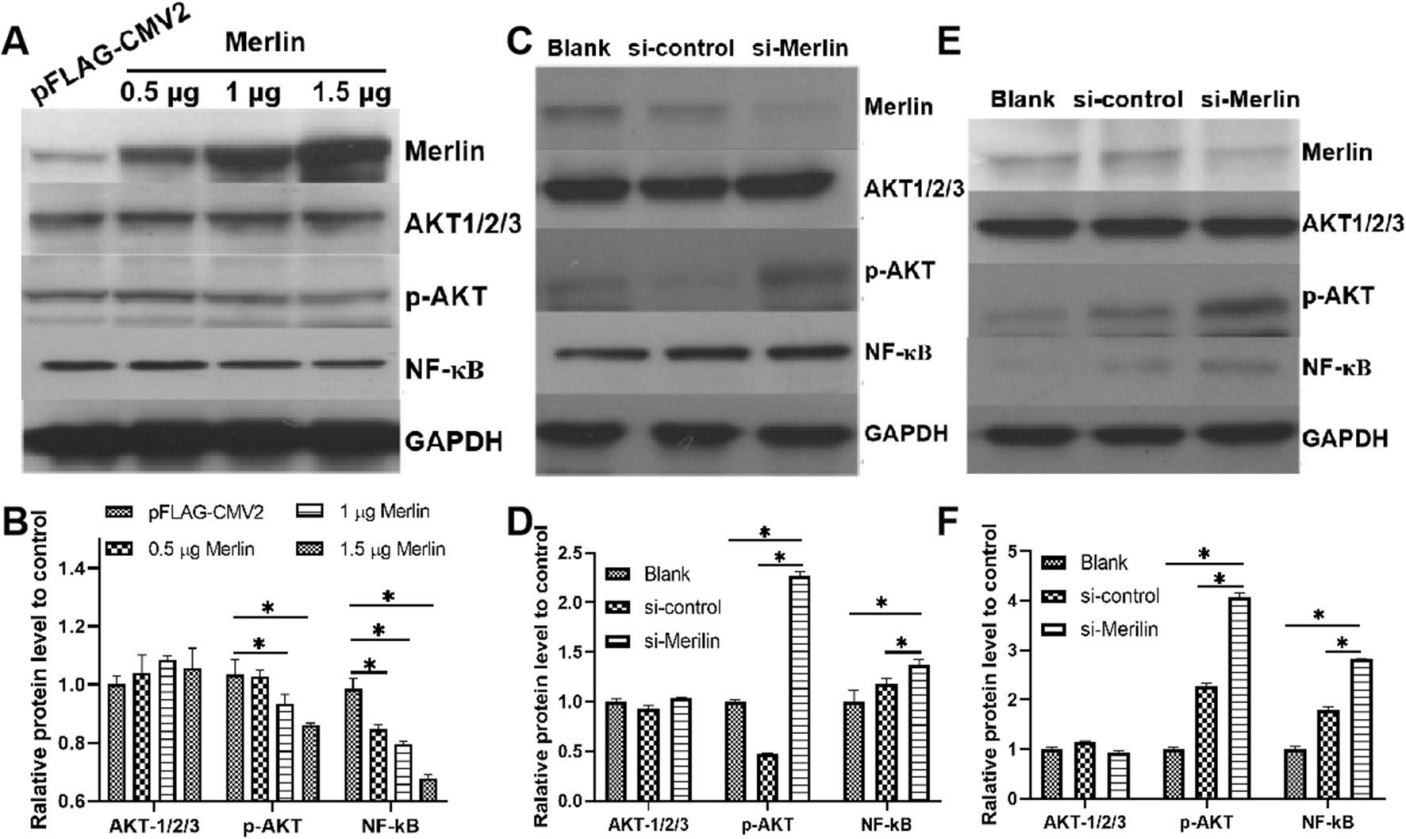
Effect of Merlin on the levels of proteins of the AKT pathway in HEK293T and Huh7.5 cells. (A and B) Huh7.5 cells were transfected with 0.5, 1, or 1.5 μg of the plasmid pFLAG-Merlin for overexpression of the Merlin protein, and plasmid pFLAG-CMV2 was used as a control. At 48 h post-transfection, the AKT1/2/3, p-AKT, and NF-κB protein levels were analyzed by western blot. (C and D) HEK293T cells were transfected with si-Merlin to silence the Merlin gene. si-control was used as a control, and PBS buffer was used as a blank control. At 48 h post-transfection, the relative levels of the AKT1/2/3, p-AKT, and NF-κB proteins were estimated. (E and F) The same procedures as in panels C and D were done using Huh7.5 cells. (*, *p* < 0.05)

Increasing the level of Merlin led to a notable decrease in the expression of p-AKT and NF-κB, while the level of total AKT1/2/3 expression remained stable (Fig. 2A and B). High expression of Merlin resulted in decreased expression of key proteins of the AKT signaling pathway. To investigate the relationship between the Hippo and AKT signaling pathways from another side, the expression of Merlin was reduced using siRNA. 50 nM siRNA against Merlin was introduced by transfection into HEK293T and Huh7.5 cells, and the relative expression levels of total AKT1/2/3, p-AKT, and NF-κB were estimated at 72 h post-transfection. As shown in Figure 2C-F, p-AKT and NF-κB protein levels increased as Merlin decreased, while AKT1/2/3 levels remained unchanged. Based on these findings, we hypothesize that NS4B mediates the AKT pathway by inhibiting Merlin, thereby influencing the Hippo signaling pathway.

### NS4B induces an increase in the number of intracellular lipid droplets

**Fig. 3:**
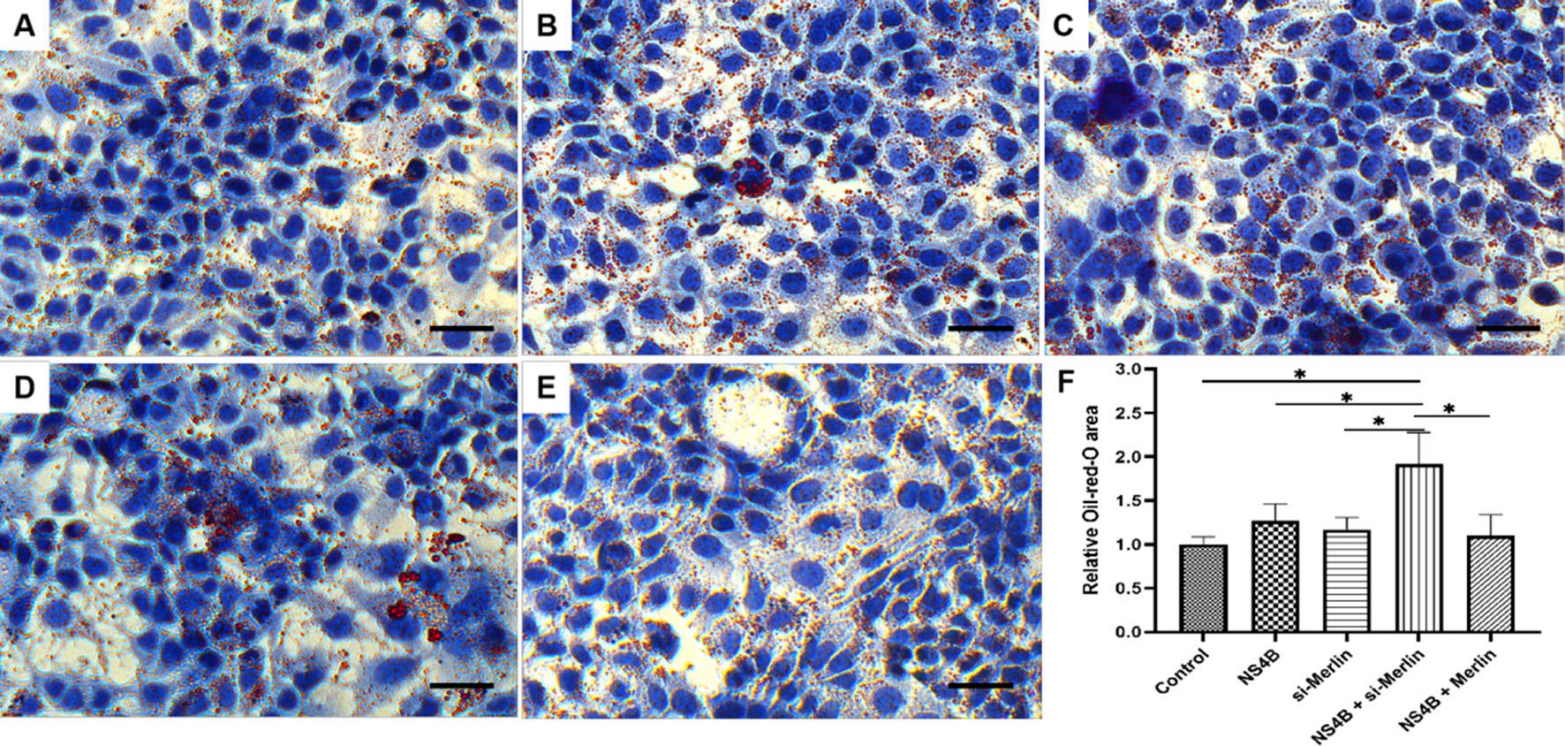
Effect of NS4B on formation of intracellular lipid droplets. (A) pCDNA3.1 + si-control. (B) pCDNA3.1-NS4B + si-control. (C) pCDNA3.1 + si-Merlin. (D) pCDNA3.1-NS4B + si-Merlin. (E) pCDNA3.1-NS4B + pFLAG-Merlin. (F) Quantification of Oil Red O areas. Values are the mean ± SD (n = 3). (*, *p* < 0.05). Scale bar = 25 μm

To investigate further whether NS4B or Merlin affects lipid metabolism in hepatocytes, we used Oil Red O tests to detect the formation of intracellular lipid droplets in Huh7.5 cells expressing different amounts of NS4B or Merlin, using cells transfected with pcDNA3.1 or si-control as controls. The number of intracellular lipid droplets increased 24 hours after transfection with the NS4B plasmid and si-Merlin compared to the control (Fig. 3A and B). Overexpression of NS4B plus si-Merlin increased the number of lipid droplets (Fig. 3D) more strongly than overexpression of NS4B or si-Merlin alone (Fig. 3B and C), suggesting that NS4B and Merlin both mediate lipogenesis in hepatocytes, but in opposite ways.

Overexpression of NS4B and Merlin together did not result in a higher level of formation of intracellular lipid droplets than overexpression of NS4B alone, indicating that NS4B may enhance the formation of intracellular lipid droplets by controlling Merlin.

### Upregulation of lipid droplet formation by NS4B is related to protein expression and transcript levels

**Fig. 4:**
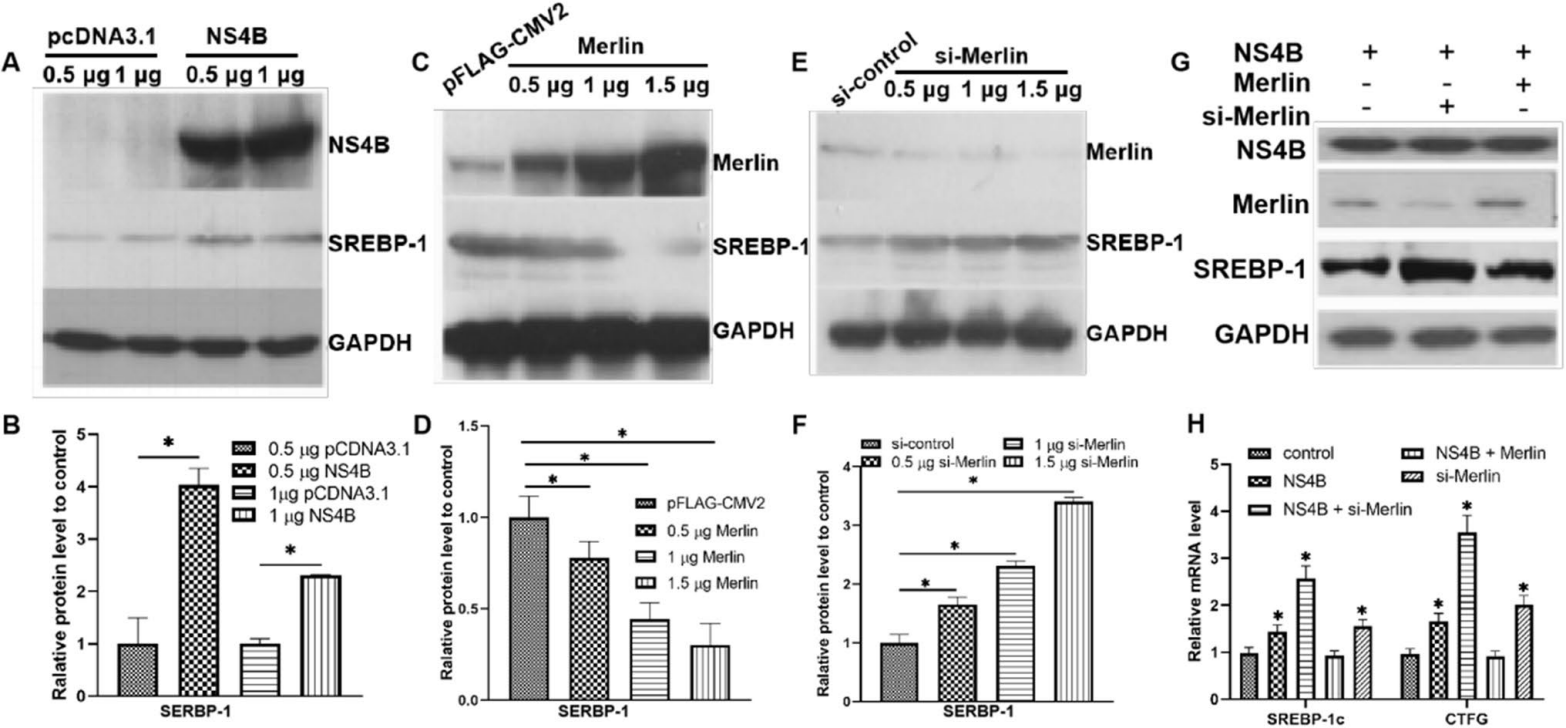
Upregulation of proteins involved in lipid droplet formation and their transcripts by NS4B and si-Merlin. (A and B) Huh7.5 cells were transfected with plasmid pcDNA3.1-NS4B to overexpress NS4B, using plasmid pcDNA3.1 as a control. The relative levels of NS4B and SERBP-1 were estimated by western blot at 48 h post-transfection. (C and D) Huh7.5 cells were transfected with 0.5, 1, and 1.5 μg of pFLAG-Merlin plasmid to overexpress Merlin, and plasmid pFLAG-CMV2 was used as a control. The levels of the Merlin and SERBP-1 proteins were analyzed by western blot at 48 h post-transfection. (E and F) Huh7.5 cells were transfected with 0.5, 1, or 1.5 μg of si-Merlin, and si-control was used as a control. The expression of Merlin and SERBP-1 was analyzed at 48 h post- transfection. (G) Huh7.5 cells were transfected with pCDNA3.1-NS4B, pFLAG-Merlin, and/or si-Merlin, and the expression of NS4B, Merlin, and SERBP-1 was analyzed. (H) The relative levels of SERBP-1 and CTFG mRNA were estimated by real-time PCR in Huh7.5 cells after transfection with pCDNA3.1-NS4B, pFLAG-Merlin, or si-Merlin. *, *p* < 0.05, compared to the control

The mechanism of lipogenesis induced by NS4B was further investigated by studying the effect of NS4B on proteins known to be related to lipid droplet formation. We studied the protein SREBP-1, a crucial endoplasmic-reticulum-binding transcription factor governing lipogenesis and lipid uptake by transfecting Huh7.5 cells with various concentrations of pCDNA3.1-NS4B, pFLAG-Merlin, or si-Merlin. The results showed that the SREBP-1 expression level was upregulated by NS4B (Fig. 4A-B) and si-Merlin (Fig. 4E-F) and downregulated by Merlin (Fig. 4C-D). When cells were cotransfected with NS4B and si-Merlin, the amount of SREBP-1 expression was significantly higher than with either alone (Fig. 4G), which is consistent with the results of the Oil Red O assays (Fig. 3).

To confirm that NS4B induces lipogenesis by affecting SREBP-1 through the YAP-Hippo and AKT pathways, the relative mRNA levels of SERBP-1 and CTFG, which is one of the direct target molecules of YAP, were studied in Huh7.5 cells transfected with pCDNA3.1-NS4B, pFLAG-Merlin, or si-Merlin. As shown in Figure 4H, Merlin was able to block the enhancement caused by NS4B, whereas both NS4B and si-Merlin were able to increase the transcription of SREBP-1C. As expected, the combination of NS4B and si-Merlin boosted the SREBP-1 mRNA level sharply. The effect of each treatment on CTFG mRNA levels was similar to that on SREBP-1 mRNA levels. We inferred that HCV NS4B triggers lipogenesis by suppressing Merlin through the Hippo and AKT pathways.

### NS4B enhances the proliferative capacity of human hepatoma carcinoma cells* in vitro*

**Fig. 5:**
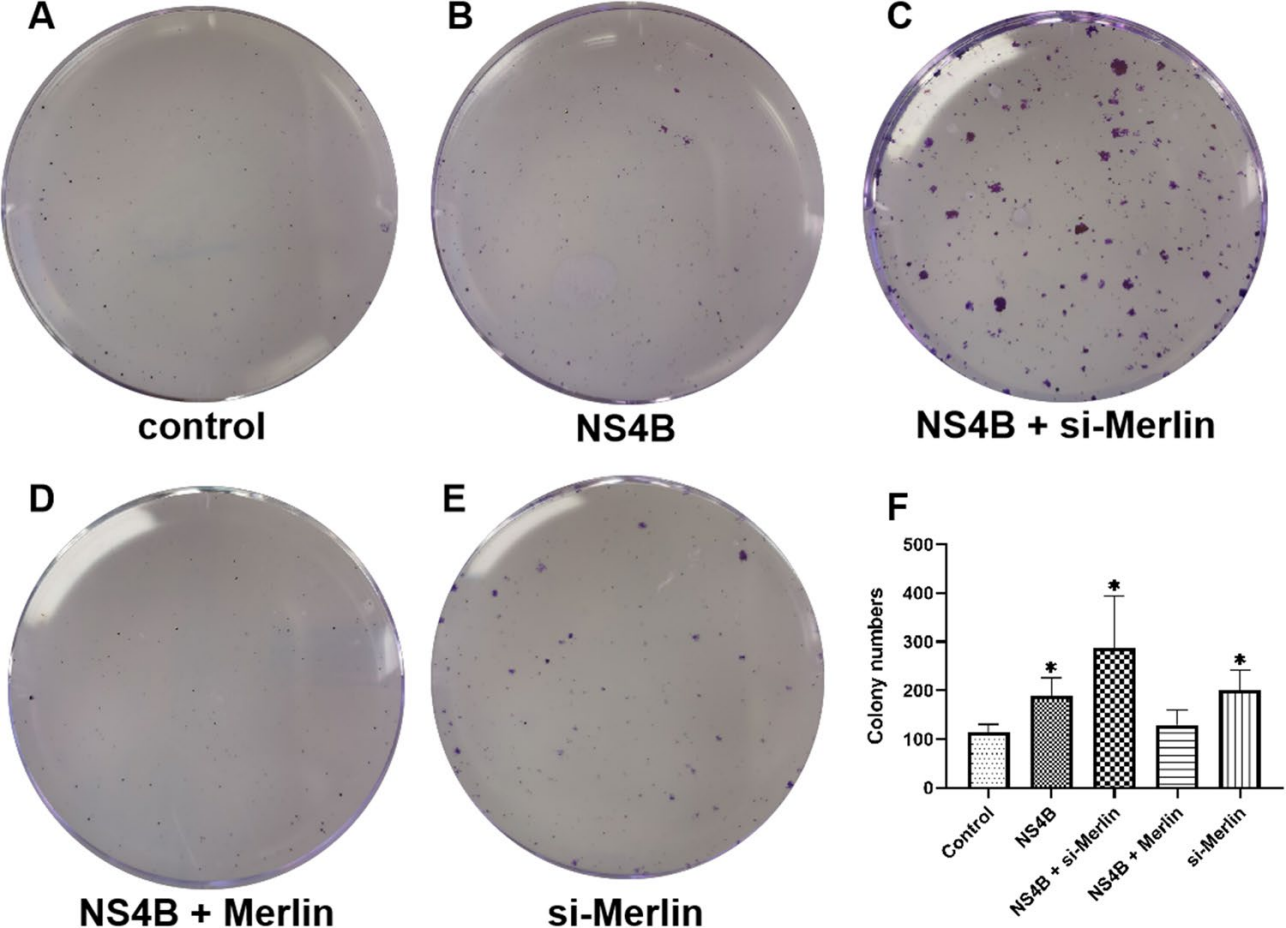
Enhancement of the *in vitro* proliferative capacity of Huh7.5 cells by NS4B. (A) pcDNA3.1 + si-control. (B) pcDNA3.1-NS4B + si-control. (C) pcDNA3.1-NS4B + si-Merlin. (D) pcDNA3.1-NS4B + pFLAG-Merlin. (E) pcDNA3.1 + si-Merlin. (F) Quantification of foci. Values are the mean ± SD (n ≥ 3) (*, *p* < 0.05, compared with the control group).

One of the primary causes of pathogenesis and intractability, including the development of liver cancer, is the capacity of HCV to interfere with the regulation of processes such as lipid metabolism in the host and triggering of lipogenesis by HCV NS4B. To investigate the effect of NS4B on the survival of human hepatoma carcinoma cells *in vitro*, colony formation assays were performed using Huh7.5 cells transfected with pCDNA3.1-NS4B, si-Merlin, or pFLAG-Merlin. Cells transfected with NS4B plus si-Merlin formed significantly more and larger colonies than other cells (Fig. 5). Cells expressing NS4B plus Merlin did not differ significantly from the controls, whereas those expressing NS4B or si-Merlin produced more colonies than the controls (Fig. 5), indicating that Merlin counteracts the enhancement induced by NS4B. The results show that NS4B enhances the capability of a single carcinoma cell to grow into a large colony via clonal expansion, potentially contributing to accelerated tumor growth.

## Discussion

The Hippo pathway, which controls cell growth, apoptosis, and proliferation and hence plays a key role in controlling tissue size, has been shown to be highly significant in mammals [15, 16]. Numerous cell types, particularly hepatocytes, have been demonstrated to be under the control of the Hippo signaling pathway [17], of which Yes-associated protein (Yap) is one of the essential downstream effectors [10]. Hippo regulates tissue growth by phosphorylation and inactivation of the transcriptional co-activator YAP. Direct phosphorylation of YAP by LATS1/2 produces phosphorylated YAP (p-YAP), which is then linked to proteins in the cytoplasm and remains there while being ubiquitinated and degraded, reducing its capacity to promote growth and prevent apoptosis [18–20]. The Merlin protein, a tumor suppressor [21], has been linked to the Hippo pathway as an upstream regulator [22, 23]. In this study, it was demonstrated that the HCV NS4B protein, which is known to induce lipogenesis, effectively downregulated the level of the Merlin protein (Fig. 1A-D). Hence, we speculated that NS4B might affect processes in cell biology in which the Hippo pathway is involved. To test this, we examined the relationship between p-YAP expression and NS4B and discovered a negative correlation between the levels of NS4B and p-YAP (Fig. 1 A-D). Consequently, we believed that the NS4B protein influences the Hippo pathway via YAP.

Previous studies [24–28] have demonstrated an association between lipid buildup and HCV infection in hepatic tissue, and hepatic steatosis occurs in between 40% and 86% of HCV-infected individuals. It has also been observed that HCV NS4B can increase the rate of lipogenesis via the AKT pathway [8]. In this study, we investigated and verified the impact of HCV NS4B on the AKT pathway. We found that NS4B upregulates the phosphorylation of AKT, which is in line with earlier reports [8]. We hypothesized that Merlin is associated with lipogenesis regulation networks, including NS4B and the AKT pathway, as HCV NS4B influences the Merlin protein level, and a relationship between Merlin and the AKT pathway has been reported [29, 30]. In this work, total AKT expression remained constant regardless of whether Merlin was overexpressed or silenced. However NF-κB and p-AKT protein levels exhibited a negative association with Merlin expression (Fig. 2). This is consistent with NS4B-induced increases in the expression of phosphorylated AKT and NF-κB (Fig. 1E, F, H and J). Therefore, the AKT pathway may be regulated upstream by the Merlin protein, and HCV NS4B may mediate the AKT pathway by modulating the Merlin protein.

Because Merlin is involved in fatty acid synthesis and lipid droplet formation is regulated via the AKT pathway [31], we investigated whether Merlin and NS4B are involved in lipid droplet formation. Our results suggest that Merlin reduces NS4B-induced lipid synthesis, while NS4B causes an increase in intracellular lipid production (Fig. 3). SREBP-1 transcription and expression levels exhibited a similar trend to lipid production (Fig. 4). SREBPs are a class of proteins that are crucial transcriptional regulators of lipid absorption and lipogenesis [32]. In mammals, there are two types of SREBPs: SREBP-1 and SREBP-2 [33, 34]. Two isoforms of SREBP-1 – SREBP-1a and SREBP-1c – can be produced from the SREBP-1 gene by transcription from distinct promoters [35, 36]. The PI3K/AKT oncogenic signaling pathway in cancer stabilizes and activates SREBP-1 [37, 38]. Additionally, it has been suggested that NS4B could stimulate fatty acid production by boosting SERBP-1 expression via the PI3K-AKT pathway [8, 26]. One of the main direct targets of YAP, PIK3CB (PIK3 catalytic subunit), has been shown recently to activate the PI3K-AKT pathway through PIK3CB and control the proliferation and survival of cardiocytes [39]. We therefore deduced that HCV NS4B might be linked to the AKT pathway through the Hippo pathway and that it is one of the crucial proteins involved in lipogenesis in hepatocytes.

Colony formation assays showed that NS4B overexpression and Merlin silencing enhanced the ability of single cancer cells to grow into large colonies. It has been observed that inactivation of the Hippo signaling pathway, which is oncosuppressive in the liver, promotes tumor formation [40]. Fatty acids may promote hepatic fibrosis by activating the transcriptional coactivator YAP1 [41]. Our observation that NS4B decreases Yap phosphorylation and Merlin protein levels, resulting in adipogenesis, suggests that NS4B might hasten tumor growth.

In conclusion, our findings suggest that HCV NS4B can cause lipogenesis by influencing the AKT signaling pathway via the Hippo-YAP pathway, thereby promoting the progression of HCV-associated diseases such as tumor growth.

## Data Availability

The data that support the findings of this study are available from the corresponding author upon reasonable request.

## References

[1] Farci P, Shimoda A, Coiana A, Diaz G, Peddis G, Melpolder Jacqueline C, Strazzera A, Chien David Y, Munoz Santiago J, Balestrieri A, Purcell Robert H, Alter Harvey J (2000). The outcome of acute hepatitis C predicted by the evolution of the viral quasispecies. Science.

[2] Hofmann S, Krajewski M, Scherer C, Scholz V, Mordhorst V, Truschow P, Schöbel A, Reimer R, Schwudke D, Herker E (2018) Complex lipid metabolic remodeling is required for efficient hepatitis C virus replication. BBA Mol Cell Biol L 1863:1041–1056. 10.1016/j.bbalip.2018.06.002. 10.1016/j.bbalip.2018.06.002 29885363

[3] Syed GH, Amako Y, Siddiqui A (2010) Hepatitis C virus hijacks host lipid metabolism. Trends Endocrin Met 21:33–40. 10.1016/j.tem.2009.07.005. 10.1016/j.tem.2009.07.005 PMC2818172 19854061

[4] Butt AA, Yan P, Simon TG, Chung RT, Abou-Samra AB (2015). Changes in circulating lipids level over time after acquiring HCV infection: results from ERCHIVES. BMC Infect Dis.

[5] Sidorkiewicz M (2021) Hepatitis C virus uses host lipids to its own advantage. Metabolites.10.3390/metabo11050273. 10.3390/metabo11050273 PMC8145847 33925362

[6] Li S, Yu X, Guo Y, Kong L (2012). Interaction networks of hepatitis C virus NS4B: implications for antiviral therapy. Cell Microbiol.

[7] Elazar M, Liu P, Rice CM, Glenn JS (2004). An N-terminal amphipathic helix in hepatitis C virus (HCV) NS4B mediates membrane association, correct localization of replication complex proteins, and HCV RNA replication. J Virol.

[8] Park C-Y, Jun H-J, Wakita T, Cheong JH, Hwang SB (2009). Hepatitis C virus nonstructural 4B protein modulates sterol regulatory element-binding protein signaling via the AKT pathway. J Biol Chem.

[9] Hu B, Xie S, Hu Y, Chen W, Chen X, Zheng Y, Wu X (2017). Hepatitis C virus NS4B protein induces epithelial-mesenchymal transition by upregulation of Snail. Virol J.

[10] Pan D (2010). The hippo signaling pathway in development and cancer. Dev Cell.

[11] Li H, Wolfe A, Septer S, Edwards G, Zhong X, Abdulkarim AB, Ranganathan S, Apte U (2012). Deregulation of Hippo kinase signaling in human hepatic malignancies. Liver Int.

[12] Tao J, Calvisi DF, Ranganathan S, Cigliano A, Zhou L, Singh S, Jiang L, Fan B, Terracciano L, Armeanu-Ebinger S, Ribback S, Dombrowski F, Evert M, Chen X, Monga SPS (2014). Activation of β-catenin and Yap1 in human hepatoblastoma and induction of hepatocarcinogenesis in mice. Gastroenterology.

[13] Xue Y, Mars WM, Bowen W, Singhi AD, Stoops J, Michalopoulos GK (2018). Hepatitis C virus mimics effects of glypican-3 on CD81 and promotes development of hepatocellular carcinomas via activation of hippo pathway in hepatocytes. Am J Pathol.

[14] Aylon Y, Oren M (2016). The hippo pathway, p53 and cholesterol. Cell Cycle.

[15] Agarwala S, Duquesne S, Liu K, Boehm A, Grimm L, Link S, König S, Eimer S, Ronneberger O, Lecaudey V (2015) Amotl2a interacts with the Hippo effector Yap1 and the Wnt/β-catenin effector Lef1 to control tissue size in zebrafish. Elife:e08201 10.7554/eLife.08201 PMC4596637 26335201

[16] Umegawachi T, Yoshida H, Koshida H, Yamada M, Ohkawa Y, Sato T, Suyama M, Krause HM, Yamaguchi M (2017). Control of tissue size and development by a regulatory element in the yorkie 3'UTR. Am J Cancer Res.

[17] Lee KP, Lee JH, Kim TS, Kim TH, Park HD, Byun JS, Kim MC, Jeong WI, Calvisi DF, Kim JM, Lim DS (2010). The Hippo–Salvador pathway restrains hepatic oval cell proliferation, liver size, and liver tumorigenesis. PNAS.

[18] Zhao B, Wei X, Li W, Udan RS, Yang Q, Kim J, Xie J, Ikenoue T, Yu J, Li L, Zheng P, Ye K, Chinnaiyan A, Halder G, Lai ZC, Guan KL (2007). Inactivation of YAP oncoprotein by the Hippo pathway is involved in cell contact inhibition and tissue growth control. Gene Dev.

[19] Dong J, Feldmann G, Huang J, Wu S, Zhang N, Comerford SA, Gayyed MF, Anders RA, Maitra A, Pan D (2007). Elucidation of a universal size-control mechanism in Drosophila and mammals. Cell.

[20] Zhao B, Li L, Tumaneng K, Wang CY, Guan KL (2010). A coordinated phosphorylation by Lats and CK1 regulates YAP stability through SCF (beta-TRCP). Gene Dev.

[21] Cui Y, Groth S, Troutman S, Carlstedt A, Sperka T, Riecken LB, Kissil JL, Jin H, Morrison H (2019). The NF2 tumor suppressor merlin interacts with Ras and RasGAP, which may modulate Ras signaling. Oncogene.

[22] Wu J, Minikes AM, Gao M, Bian H, Li Y, Stockwell BR, Chen ZN, Jiang X (2019). Intercellular interaction dictates cancer cell ferroptosis via NF2-YAP signalling. Nature.

[23] Wu J, Minikes AM, Gao M, Bian H, Li Y, Stockwell BR, Chen ZN, Jiang X (2019). Publisher correction: intercellular interaction dictates cancer cell ferroptosis via NF2-YAP signalling. Nature.

[24] Ferguson D, Zhang J, Davis MA, Helsley RN, Vedin LL, Lee RG, Crooke RM, Graham MJ, Allende DS, Parini P, Brown JM (2017). The lipid droplet-associated protein perilipin 3 facilitates hepatitis C virus-driven hepatic steatosis. J Lipid Res.

[25] Felmlee DJ, Hafirassou ML, Lefevre M, Baumert TF, Schuster C (2013). Hepatitis C virus, cholesterol and lipoproteins–impact for the viral life cycle and pathogenesis of liver disease. Viruses.

[26] Kim KH, Hong SP, Kim K, Park MJ, Kim KJ, Cheong J (2007). HCV core protein induces hepatic lipid accumulation by activating SREBP1 and PPARgamma. Biochem Bioph Res Co.

[27] Harada R, Kimura M, Sato Y, Taniguchi T, Tomonari T, Tanaka T, Tanaka H, Muguruma N, Shinomiya H, Honda H, Imoto I, Sogabe M, Okahisa T, Takayama T (2018). APOB codon 4311 polymorphism is associated with hepatitis C virus infection through altered lipid metabolism. BMC Gastroenterol.

[28] Lavie M, Dubuisson J (2017). Interplay between hepatitis C virus and lipid metabolism during virus entry and assembly. Biochimie.

[29] Okada M, Wang Y, Jang SW, Tang X, Neri LM, Ye K (2009). Akt phosphorylation of merlin enhances its binding to phosphatidylinositols and inhibits the tumor-suppressive activities of merlin. Can Res.

[30] Tang X, Jang SW, Wang X, Liu Z, Bahr SM, Sun SY, Brat D, Gutmann DH, Ye K (2007). Akt phosphorylation regulates the tumour-suppressor merlin through ubiquitination and degradation. Nat Cell Biol.

[31] Stepanova DS, Braun L, Chernoff J (2018) A new concept in NF2 pharmacotherapy: targeting fatty acid synthesis. Oncoscience 5:126–127. 10.18632/oncoscience.417 10.18632/oncoscience.417 PMC6049319 30035161

[32] Horton JD, Goldstein JL, Brown MS (2002). SREBPs: activators of the complete program of cholesterol and fatty acid synthesis in the liver. J Clin Invest.

[33] Brown MS, Goldstein JL (1997). The SREBP pathway: regulation of cholesterol metabolism by proteolysis of a membrane-bound transcription factor. Cell.

[34] Osborne TF, Espenshade PJ (2009). Evolutionary conservation and adaptation in the mechanism that regulates SREBP action: what a long, strange tRIP it's been. Gene Dev.

[35] Horton JD, Shimomura I, Ikemoto S, Bashmakov Y, Hammer RE (2003). Overexpression of sterol regulatory element-binding protein-1a in mouse adipose tissue produces adipocyte hypertrophy, increased fatty acid secretion, and fatty liver. J Biol Chem.

[36] Yokoyama C, Wang X, Briggs MR, Admon A, Wu J, Hua X, Goldstein JL, Brown MS (1993). SREBP-1, a basic-helix-loop-helix-leucine zipper protein that controls transcription of the low density lipoprotein receptor gene. Cell.

[37] Porstmann T, Griffiths B, Chung YL, Delpuech O, Griffiths JR, Downward J, Schulze A (2005). PKB/Akt induces transcription of enzymes involved in cholesterol and fatty acid biosynthesis via activation of SREBP. Oncogene.

[38] Yecies JL, Zhang HH, Menon S, Liu S, Yecies D, Lipovsky AI, Gorgun C, Kwiatkowski DJ, Hotamisligil GS, Lee CH, Manning BD (2011). Akt stimulates hepatic SREBP1c and lipogenesis through parallel mTORC1-dependent and independent pathways. Cell Metab.

[39] Lin Z, Zhou P, von Gise A, Gu F, Ma Q, Chen J, Guo H, van Gorp PR, Wang DZ, Pu WT (2015). Pi3kcb links Hippo-YAP and PI3K-AKT signaling pathways to promote cardiomyocyte proliferation and survival. Circ Res.

[40] Liu Y, Wang X, Yang Y (2020). Hepatic Hippo signaling inhibits development of hepatocellular carcinoma. Clin Mol Hepatol.

[41] Salloum S, Jeyarajan AJ, Kruger AJ, Holmes JA, Shao T, Sojoodi M, Kim MH, Zhuo Z, Shroff SG, Kassa A, Corey KE, Khan SK, Lin W, Alatrakchi N, Schaefer EAK, Chung RT (2021). Fatty acids activate the transcriptional coactivator YAP1 to promote liver fibrosis via p38 mitogen-activated protein kinase. Cell Mol Gastroenterol Hepatol.

